# The Effect of a Personalized Oral Health Education Program on Periodontal Health in an At-Risk Population: A Randomized Controlled Trial

**DOI:** 10.3390/ijerph18020846

**Published:** 2021-01-19

**Authors:** Eman S. Almabadi, Adrian Bauman, Rahena Akhter, Jessica Gugusheff, Joseph Van Buskirk, Michelle Sankey, Janet E. Palmer, David J. Kavanagh, Gregory J. Seymour, Mary P. Cullinan, Joerg Eberhard

**Affiliations:** 1 School of Dentistry, Faculty of Medicine and Health, The University of Sydney, Camperdown, Sydney, NSW 2006, Australia or emabadi@taibahu.edu.sa (E.S.A.); rahena.akhter@sydney.edu.au (R.A.); 2 Pediatric Dentistry and Orthodontics Department, College of Dentistry, Taibah University, Al Madinah Al Munawara 41477, Saudi Arabia; 3 Prevention Research Collaboration, Charles Perkins Centre, The University of Sydney, Sydney, NSW 2006, Australia; adrian.bauman@sydney.edu.au (A.B.) jessica.gugusheff@health.nsw.gov.au (J.G.); joseph.vanbuskirk@health.nsw.gov.au (J.V.B.); 4 School of Public Health, The University of Sydney, Sydney, NSW 2006, Australia; 5 Oral Health Clinic, Logan Hospital, Meadowbrook, Queensland, QLD 4131, Australia; msankey@live.com.au; 6 School of Dentistry, The University of Queensland, Herston, QLD 4072, Australia; j.palmer@uq.edu.au (J.E.P.); g.seymour@uq.edu.au (G.J.S.); m.cullinan@uq.edu.au (M.P.C.); 7 Centre for Children’s Health Research and School of Psychology & Counselling, Queensland University of Technology (QUT), South Brisbane, QLD 4072, Australia; david.kavanagh@qut.edu.au

**Keywords:** oral health, randomized controlled trial, periodontal health, gingival bleeding, personalized education

## Abstract

While periodontal disease is associated with many risk factors, socioeconomically disadvantaged communities experience the highest disease burden. The aim of this study was to evaluate the effectiveness of a personalized oral health education program, in combination with routine dental treatment, in participants from a low socioeconomic community. We used a randomized, controlled, examiner-blinded clinical trial. A total of 579 participants (aged 18–60 years) were randomly grouped: the intervention group (n = 292) received a personalized oral health education program in combination with routine dental care and the control group (n = 287) received routine dental care. All participants were assessed for improvement in oral health care behaviors, dental plaque, and periodontal status at baseline, 12 months, and 24 months. We found a significant drop (*p* < 0.001) in the plaque indices, Periodontal Probing Depths (PPD) and Bleeding on Probing (BOP) between baseline and the 12-month follow-up for both groups. For BOP, the number of sites positive was significantly different between baseline and the 24-month follow-up (*p* = 0.037). No differences were found between the two groups for any evaluated clinical outcome. The personalized oral health education program used in the current study did not appear to add significant improvement to clinical outcomes of periodontal health compared with routine restorative dental care per se.

## 1. Introduction

Oral health refers to a healthy state of the oral cavity and related tissues that enables an individual to chew, speak, and confidently socialize without pain, discomfort, and or embarrassment [1]. The maintenance of good oral health is integral to maintaining an individual’s overall health and is a fundamental right for everyone [2,3,4]. Poor oral health has a profound effect on the quality of life and contributes to poor nutrition, lost attendance at school or work, and impacts life-related activities as a result of pain and suffering [5]. Periodontal diseases, dental caries, and the consequent tooth loss can all be considered as the common clinical indicators of poor oral health [6].

Periodontal diseases are the most common oral pathological conditions [7] and approximately 90% of the world’s population is affected by chronic inflammatory periodontal disease, which includes both gingivitis and periodontitis [8]. It is caused by bacteria in dental plaque which induce a progressive inflammatory response that is followed by the destruction of the tooth supporting tissues [9]. Dental caries is also a common oral condition caused by bacterial deposits accumulating on tooth surfaces [10]. A large body of evidence has demonstrated that poor oral hygiene, a diet high in carbohydrates, salivary status, medical history, genetic factors, and socioeconomic status determines the risk for dental caries [11,12]. In addition smoking, alcohol consumption, and psychological factors, are associated with chronic inflammatory periodontal disease [13,14]. Effective oral self-care behavior (i.e., twice a day brushing with fluoride toothpaste combined with flossing once per day) is fundamental for the successful prevention and treatment of both diseases [15].

Oral health education programs aim to improve the oral health of a targeted population by implementing oral health care behavioral changes [16]. Several published studies have shown that such programs are effective in both children and adults for improving knowledge and attitudes towards dental care to lower the burden of the oral diseases [17,18]. Oral health promotion should preferentially target low socioeconomic populations, as they experience the greatest burden of poor oral health [19,20]. However, oral health programs in low socioeconomic populations are rare and generally limited to follow up periods of 6 months and do not allow the assessment of the long-term effects of an educational program [21,22]. A major flaw of research into educational programs in this group is that they address preventive behavioral changes in participants with no dental treatment needs [23,24]. Furthermore, a stand-alone educational program in population with existent dental treatment needs does not reflect standard oral health care. An oral health educational program accompanied by the treatment of periodontal disease and/or dental caries, if necessary, might have a significant improvement of oral health in such population. Therefore, the disconnect between an educational program and treatment does not allow conclusions to be drawn regarding the benefits of an educational program in a real-world clinical setting.

In health psychology and behavioral science, motivational interviewing (MI) has been developed to enhance individual readiness for a positive behavioral change and strengthen the individual’s commitment to change [25]. This method focuses on preparing the individual for behavior change by exploring and resolving ambivalence about change and making their own decision about how and why to proceed [26]. Studies using the MI in dental care have provided promising results in improving oral health behavior [27,28]. However, it remains uncertain whether this kind of intervention is effective for a group of patients from a low socioeconomic community.

Based on these points, this randomized controlled trial aimed to determine the effectiveness of a personalized oral health education program (OHEP) based on MI, in combination with routine dental treatment in participants from a low socioeconomic community. The primary outcomes investigated in this study included Periodontal Probing Depth (PPD) and Bleeding on Probing (BOP) over a 24-month follow-up period.

## 2. Materials and Methods

### 2.1. Study Design, Registration, and Ethical Approvals

This study was a randomized, controlled, examiner-blinded clinical trial with two parallel arms. It was registered on the Australian New Zealand Clinical Trials Registry (ACTRN12605000607673). The study was conducted in accordance with the Declaration of Helsinki, and the ethical approval was obtained from the human ethics committees of The University of Queensland (2002000423) and Princess Alexandra Hospital (2002/074).

### 2.2. Setting

The study was conducted at the Logan Hospital Oral Health Care Clinics, Queensland, Australia. The hospital dental clinic is accessible to all residents in the Logan-Beaudesert area. Comprehensive general dentistry is provided by the clinic but the waiting list at the time was over 4 years for non-emergency general dentistry. At that time, there were 4 general dentists and 2 oral health therapists. The participants were enrolled between March 2003 and June 2006, and all data collection was completed by March 2008, which included baseline data collection, and two follow-up assessment periods. According to the Australian Bureau of Statistics, the Index of Relative Socio-Economic Disadvantage (IRSD) score for this area is 955, well (4.5%) below the average scores for Australia (1000). This highlights the relatively lower socioeconomic status profile of the Logan population [29].

### 2.3. Study Population

The subjects were recruited from the existing public patient waiting list for routine dental care and those attending for emergency dental care. The following inclusion criteria were applied: (1) age ranging from 18–60 years; (2) a minimum of 12 teeth; (3) eligible for public dental services; (4) no requirement for prophylactic antibiotic cover for dental procedures; and (5) consent to be available for the follow-up assessments. Informed consent was obtained from all subjects after being given a written and verbal explanation of the study. The intervention is described according to the CONSORT (Consolidated Standards of Reporting Trials) statement [30].

### 2.4. Sample Size and Power Calculation

The sample size calculation was based on a previous longitudinal study carried out at The University of Queensland, Australia [31]. Our power calculation showed a sample size of 250 per group was sufficient to show a reduction of 60% in plaque and gingivitis scores with a power of 80%.

### 2.5. Intervention

During the recruitment, the dentists (T.W., C.L., J.Y., S.L.) and a research nurse (V.A.) collected all baseline and the follow-up assessment data, including demographic data, medical history, and physical parameters. All participants had a baseline oral examination that included PPD and BOP at 6 sites per tooth, and a plaque index. After the baseline visit, participants were randomized to either the intervention or control group. Randomization was performed using computer-generated random allocation in sealed envelopes prepared by an independent person at the start of the study.

Participants in the intervention group were referred to the Personalized Oral Health Education Programme (OHEP) prior to routine dental treatment. The OHEP was tailored to each participant’s oral health needs and completed over a maximum of four (45 min) visits. The OHEP was completed according to the following components. At the initial visit, each participant received tailored oral health instruction. It accounted for the participant’s current oral health status and risk factors for periodontal disease. This unique personalized oral health education program was tailored to each participant’s oral health needs, and it was based on motivation interviewing [25] to prompt and strengthen motivation for oral health behavioral change. The OHEP provided by two oral health therapists/hygienists who received training in MI method by a specialized clinical psychologist (J.C.), drawing on materials co-developed with (D.J.K.). It also included written and oral information about the effects of smoking, alcohol, and diet on oral health. In addition, a healthy diet was promoted, particularly reducing the consumption of sugars and increasing consumption of fruits and vegetables, in accordance with Dietary Guidelines for Australian Adults [32]. At the second visit, the participants received oral hygiene instruction and the importance of their efforts regarding daily oral health care was emphasised. The toothbrushing (Bass Technique) and interdental cleaning in a layered approach were also demonstrated at this visit. All information was continuously reinforced throughout the treatment phase, and the program was supplemented by the monthly provision of toothbrushes and toothpaste. The third visit consisted a full mouth debridement by quadrant (under local anesthesia where required), using a combination of hand and ultrasonic scalers.

At the last visit, the focus was on caries control by removing any overhanging restorations or defective restorative margins when necessary. At each visit, the participants were motivated to adhere to oral self-care recommendations to attain optimal oral health. Participants who wanted to change behaviors affecting their oral health were encouraged to set specific goals for each behavioral target, shown how to anticipate high-risk situations and how to apply problem-solving to these situations, plan to use pleasurable substitute activities, and acquire assertion skills to reduce the risk of lapses due to social pressure. The participants in the control group waited for 4 weeks before receiving routine restorative dental care that included dental scaling and oral hygiene advice at a single appointment, but no personalized oral health education program was provided to this group. The participants in both groups were contacted at monthly intervals to update any change of address. 

### 2.6. Oral Care Management Questionnaire

A structured questionnaire was used to assess the confidence to maintain oral care behaviors with respect to brushing teeth twice daily or flossing once per day according to the standard dental recommendation. The participants were asked to respond to the following statement: “please rate your degree of confidence to be able to maintain the following behaviors for the next 6 weeks” Each item in the questionnaire was provided on a 10-point interval percent scale ranging from 0 to 100. (0 = “Not at all confident” to 100 = “completely confident”), which was adapted from the oral care self-efficacy scale previously described [33]. 

### 2.7. Oral Assessment

Oral assessments were recorded by four dentists who were trained and calibrated by an experienced calibrated examiner (M.P.C.) for periodontal measures at The University of Queensland School of Dentistry. The intervention and control groups were assessed at baseline (T_0_), at the first follow-up (T_1_), (12 months after baseline), and the second follow-up (T_2_), (24 months after baseline). PPD and BOP were recorded at six sites per tooth (disto-buccal, buccal, mesio-buccal, disto-lingual, lingual, and mesio-lingual) using a light probing force of approximately 20 g at an angulation parallel to the long axis of the tooth using a William’s probe calibrated in millimeters (PW #30, Hu-Friedy, Chicago, IL, USA). The presence of BOP was recorded immediately after the PPD measurement.

For the plaque index, a modification of the Silness-Löe Index was used to provide a better level of reproducibility. A dichotomous score (combining scores 0 and 1 and scores 2 and 3) was used instead of the graded score [34]. The modified Silness–Löe Index was recorded on the buccal and lingual surfaces of all teeth except for the third molars, using a “yes” or “no” decision for the presence or absence of visible plaque. The percentage of tooth surfaces showing plaque in relation to all tooth surfaces examined was calculated.

### 2.8. Statistical Analysis

A complete case analysis was conducted on all data and only those participants with values across all time points were included in each analysis. A complete case analysis approach was selected due to the high rates of missing data. To control for correlation within participants across time points, generalized estimating equation (GEE) regression was used, grouped by the participant, with an auto-regressive correlation structure. The GEE investigating confidence to either brush teeth twice daily or floss once daily was modeled using a normal distribution. To assess the proportion of sites with either BOP or plaque, GEE was modeled using a Poisson distribution with an offset of the natural logarithm of the total number of sites. This allowed for the modeling of a percentage of sites with either BOP or plaque. Finally, a GEE model using a binomial distribution was used to assess the odds of a participant having a PPD of 5 mm or greater. The GEE model was used to examine the overall effect of the intervention on the groups, time and the interaction between the two. All statistical analyses were performed using R version 3.5.3.

## 3. Results

**Figure 1:**
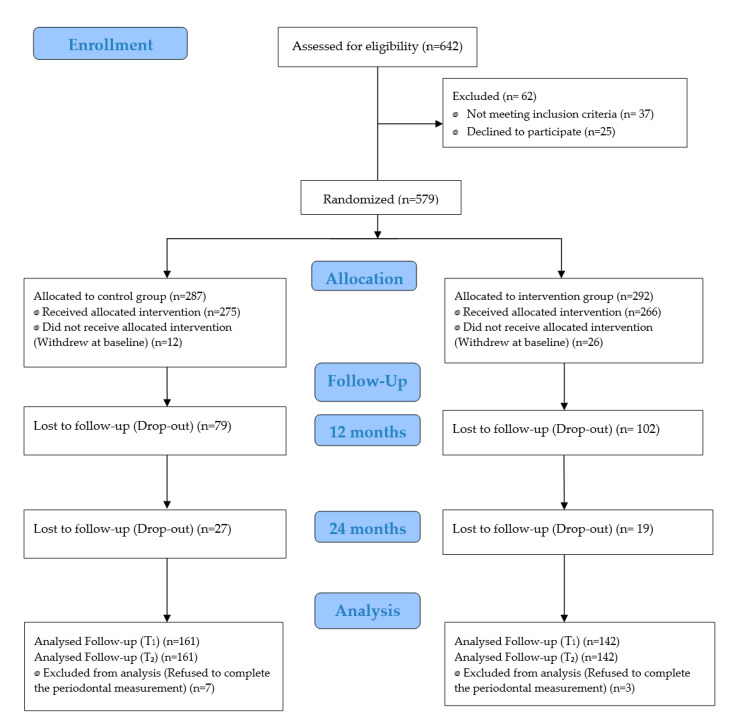
CONSORT (Consolidated Standards of Reporting Trials) flow chart of participant recruitment.

Among 579 participants enrolled in the study, 287 participants were randomized to the control group and 292 to the intervention group. Over the study period, there were 150 dropouts from the intervention group and 126 from the control group (Figure 1). A comparison of demographic characteristics and outcome variables, including PPD and BOP at baseline, revealed no significant differences between those who remained in the trial and those who dropped out, suggesting that the data are missing completely at random.

At the beginning of the study, assessment follow-up visits were proposed at 12 and 24 months from the baseline visit. However, because of the large number of participants and variations in time between follow-up visits, there were time spans of 12–16 months for the T_1_ follow-up and 24–36 months for the T_2_ follow up. A sensitivity analysis of variability in the follow-up times indicated that the outcomes were not associated with the length of time between assessments.

Demographic and baseline characteristics of participant data are presented in Table 1. The randomization was successful, as there was no statistically significant difference between the groups in any demographic or baseline characteristic (*p*-value not shown in the table). 

### 3.1. Confidence to Manage Oral Care

The confidence in maintaining tooth brushing and flossing is depicted in Table 2. For the confidence to brush the teeth twice per day, the statistical analysis showed no significant differences between the groups or between baseline and the follow-up assessments. However, a trend was observed (Z = 3.36; *p* = 0.07) that participants in the intervention group demonstrated more confidence in brushing their teeth twice a day compared with participants in the control group, across all time points. The subjects in the intervention group were more confident to clean the interdental area once per day over the entire study period compared with participants in the control group (*p* = 0.01). However, there was an overall decline of the mean value for confidence to clean interdentally once per day from 86.24% (95% CI: 81.13, 91.34) at baseline to 67.58% (95% CI: 60.79, 74.37) at the T_2_ follow-up assessment in the intervention group that did not reach statistical significance (*p* = 0.06).

### 3.2. Dental Plaque

The plaque index significantly dropped (*p* < 0.001) between baseline and the T_1_ follow-up for the intervention from 77.17% (95% CI: 71.99, 82.73) to 69.02% (95% CI: 64.18, 74.22) and the control group from 80.48% (95% CI: 76.04, 85.17) to 72.31% (95% CI: 67.93, 76.98) (Table 3). This significant difference was not observed between baseline and the T_2_ follow up. No overall differences in plaque indices were observed between groups at baseline and follow-up assessments (Z = 0.84; *p* = 0.36).

### 3.3. Periodontal Status

Table 3 shows estimated marginal means for the proportion of participants with a PPD ≥ 5 mm, and the percentage of sites positive for BOP for both groups at T_0_ and the follow-up visits T_1_ and T_2_. For PPD, the statistical analysis showed a significant reduction between baseline and the T_1_ follow-up in the intervention and control groups (*p* < 0.001). The percentage of sites positive for BOP was significantly lower between the baseline and both the following up visits T_1_ (*p* < 0.001) and T_2_ (*p* = 0.04) for both groups. No significant differences were found between the intervention and control groups at any time point (Table 3).

## 4. Discussion

This study was undertaken to investigate the effect of an oral health education program as an adjunct to standard routine restorative care on clinical parameters of periodontal disease. The central finding of this study was that although the clinical parameters of BOP and PPD improved in both study groups over the study period, there was a lack of additive effects for participants allocated to the personalized oral health education program. This may be related to the fact that low socioeconomic status can influence the effectiveness of health behavior change in any interventions [35].

The pragmatic approach utilized for the current study and the long-term follow-up are strengths of the study that enable generalization of the study outcomes to different populations and different systems of oral health care delivery. There are several limitations in this study. A low level of participant retention was found in the study; however, dropout rates of 46% were observed in other oral health education programs conducted in low socioeconomic populations over a follow-up period of 3 years [36]. Also, data completion was in 2008, and it may be speculated that a more recent study would provide different outcomes due to changes in lifestyle. However, the oral health status in Australia has only marginally changed over the last decade and the funding model is the same as in 2008 [37]. Furthermore, the study did not provide data on the fidelity of the delivered intervention to the spirit of motivation enhancement, although the intervention was manualized and participating staff received training in the intervention prior to its use in the trial. A further limitation was the inability to carry out reliability examinations. The examining dentists were trained and calibrated, but it was not possible, nor feasible for patients to take time away from work for multiple reliability examinations with no obvious outcome benefit for them. Equally, in an extremely busy public clinic with patients queuing from 6:00 a.m. for emergency dental treatment and where the waiting list for routine treatment was over 4 years it was not possible to bring back participants for reliability measures, especially when treatment targets needed to be met. This was mitigated by the fact that any slight variations would have minimal effect on the overall outcome of the study.

The population investigated in the current study showed several indicators of low socioeconomic background, including low educational attainment and low numbers of full-time employment. These socio-demographic characteristics are also associated with indicators for poor general health, including a higher number of overweight or obese participants, and a higher proportion of smokers and excessive alcohol consumption compared with the general population [29]. It has been reported that residents of the region in which this study was conducted have poorer oral health compared with the overall Australian population, indicating that the area should have priority in the implementation of oral health education programs [38].

The current study utilized the motivational interviewing concept to promote behavioral change in combination with standard routine restorative care. However, the combination of the oral health educational program and dental treatment carries high plausibility as a potential “best practice” and represents the key context for the delivery of education in a teachable moment, where concern about dental health may be at its height. It, therefore, represents the most desirable experimental way to estimate the contribution of a behavioral intervention to clinical outcomes. There are mixed results in the studies that implemented the motivational interviewing method on oral health behavior interventions [28,39,40]. This results heterogeneity might depend on the duration, components, and the number of sessions delivered in each study.

Our study indicated that the personalized oral health education program was not followed by significant behavioral changes in the intervention group compared with the control group. The intervention was tailored to the individual participant readiness for behavior change such that a number of participants in the intervention group may not have been ready to make the necessary oral health behavioral change. It also should be noted that participants were assessed using the confidence to manage oral care and that might be not adequate for evaluation as it relies on each patient’s sense of self-efficacy. Also, as the long waiting times for dental services have a negative effect on those participants’ oral health; it is also noteworthy to consider that this might affect the behavior change. Furthermore, evidence from reviewing oral health promotion in dental settings suggests that the effectiveness on behavior change might be attributed to the relationship and understanding between the receiver and the sender of the oral health promotion [41]. Thus, a future oral health education program that includes a team of oral health professionals trained in health psychology, and how to remove any barriers that prevent participants from believing and committing to the health behavior change, should be considered. McClure et al. (2019), also did not find significant differences between intervention and control groups in self-efficacy to brush teeth twice daily at 6 months after an oral health behavioral intervention [42]. By contrast, other studies reported a positive change of oral self-care behaviors after different oral health educational programs [43,44]. This difference may be explained at least in part by the shorter follow-up period of 2 months in these studies, which may impact the oral care behavior outcomes.

The limited effect of the educational program on oral health behaviors is supported by no difference in the capacity to remove dental plaque between the two groups, as measured by the amount of dental plaque on tooth surfaces. However, for both groups, the plaque indices improved over the initial period of the study. The initial reduction in the amount of dental plaque in both groups can be explained, in part, by the dental treatment itself and better accessibility of tooth surfaces following dental treatment. It is also well documented in the literature that participants in clinical trials improve certain behaviors in awareness of a follow-up assessment [45]. A similar reduction of plaque deposits in the intervention and control groups in the present study throughout the initial study period is in agreement with reports demonstrating a reduction of plaque indices 6 months after a dental health education program [36].

Similarly, both the intervention and the control groups showed a significant improvement in PPD and BOP, which is more likely due to the initial reduction in plaque seen in both groups. However, the improvement of PPD was restricted to the initial follow-up time period of 12 to 16 months, while the reduction of BOP was observed beyond this time period. This observation is in accordance with the observations by Clarkson et al. (2009) and Ramsay et al. (2018) who were not able to demonstrate a significant difference in periodontal clinical outcomes between personalized oral health advice compared with routine oral health advice in a multicenter dental practice based 3-year trial [46,47]. By contrast, studies that have investigated the effect of different oral health education programs in school children found a superior effect of an oral health education program in favor of the experimental group when compared with the control group [48,49,50]. However, the comparison of studies is constrained by different study designs and target populations, diverse oral health education programs, and observation periods. The cumulative evidence of these studies, however, highlights the importance of regular maintenance appointments after the first intervention period to maintain any improvements in periodontal parameters.

## 5. Conclusions

In conclusion, the long-term beneficial effect of the personalized oral health education program used in the current study did not appear to significantly improve clinical outcomes of periodontal health compared with standard dental treatment alone. Both groups showed significant improvement in PPD and BOP at 12 months and BOP at 24 months. A future oral health education program based on behavioral science and involving a team of skilled oral health professionals trained in health psychology might obtain better behavioral changes in communities of low socioeconomic status.

## Figures and Tables

**Table 1:** Demographic and baseline characteristics of participants.

Characteristic		Control Group, n (%) (n = 287)	Intervention Group, n (%) (n = 292)	TOTAL, n (%) (n = 579)
Gender	Male	101 (35.19)	100 (34.25)	201 (34.72)
	Female	186 (64.81)	192 (65.75)	378 (65.28)
Age, median (range), (Years)		42.50 (52)	43 (49)	43 (55)
Marital status	Married	140 (48.78)	145 (49.65)	285 (49.22)
	Single	75 (26.13)	81 (27.74)	156 (26.94)
	Widowed or separated/divorced	72 (25.09)	66 (22.60)	138 (23.83)
Ethnicity ‡	Asian	11 (3.83)	12 (4.11)	23 (3.97)
	Caucasian	269 (93.73)	273 (93.49)	542 (93.61)
	Indigenous	6 (2.09)	7 (2.39)	13 (2.24)
Educational level †	Primary School	25 (8.71)	37 (12.67)	62 (10.71)
	High School	131 (45.64)	135 (46.23)	266 (45.94)
	Certificate/Diploma/Trade	90 (31.36)	75 (25.68)	165 (28.50)
	University Degree	20 (6.97)	24 (8.22)	44 (7.60)
	Other	19 (6.62)	20 (6.85)	39 (6.74)
Occupation ‡	Full time employed	71 (24.74)	63 (21.58)	134 (23.14)
	Part Time/Casually employed	45 (15.68)	58 (19.86)	103 (17.79)
	Unemployed	19 (6.62)	19 (6.51)	38 (6.56)
	Full or part time studying	151 (52.61)	152 (52.05)	303 (52.33)
Medical Conditions	Cardiovascular diseases	51 (17.77)	61 (20.89)	112 (19.34)
	Diabetes	22 (7.66)	24 (8.22)	46 (7.94)
Smoking Status	Smoker	110 (38.33)	123 (42.12)	233 (40.2)
	Non-Smoker	177 (61.67)	169 (57.88)	346 (59.76)
Alcohol consumption ‡	Non-Drinker	87 (30.31)	72 (24.66)	159 (27.46)
	Light-Drinker	143 (49.82)	161 (55.14)	304 (52.60)
	Heavy/medium drinker	57 (19.86)	58 (19.86)	115 (19.86)
Weight status (BMI) ¥	Underweight	13 (4.53)	13 (4.45)	26 (4.49)
	Healthy weight	89 (31.01)	88 (30.14)	177 (30.57)
	Overweight	84 (29.27)	88 (30.14)	172 (29.71)
	Obese	96 (33.45)	102 (34.93)	198 (34.20)

* Data are N (%) unless otherwise specified. † Three participants did not provide Education information. ‡ One participant did not provide this information. ¥ Six participants did not undergo body mass index measurement.

**Table 2:** Estimated marginal means and 95% confidence interval (95% C.I.) for degree of confidence at being able to maintain tooth brushing and flossing for the two-study groups at the baseline and the two follow up visits.

	Baseline (T_0_)		Follow-Up (T_1_)		Time and Intervention Effects (T_1_ vs. T_0_)	Follow-Up (T_2_)		Time and Intervention Effects (T_2_ vs. T_0_)	Overall Effect of Intervention *p*-Value
Degree of Confidence	Control (n = 161)	Intervention (n= 1 42)	Control (n = 161)	Intervention (n = 142)		Control (n = 161)	Intervention (n = 142)		
**Brush twice per day, % scale**	82.28 (77.67–86.89)	88.02 (83.96–92.08)	82.30 (77.74–86.85)	84.93 (80.37–89.49)	Time Z = 7.46, *p* = 0.99	82.18 (77.54–86.83)	81.75 (76.50–87.00)	Time Z = 0.001, *p* = 0.97	0.07
					Intervention × Time Z = 0.83, *p* = 0.36			Intervention × Time Z = 2.44, *p* = 0.12	
**Floss once per day, % scale**	75.01 (68.96–81.06)	86.24 (81.13–91.34)	68.55 (62.28–74.82)	75.70 (69.69–81.71)	Time Z = 2.80, *p* = 0.90	66.12 (59.28–72.96)	67.58 (60.79–74.37)	Time Z = 5.54, *p* = 0.02	0.01
					Intervention × Time Z = 0.67, *p* = 0.41			Intervention × Time Z = 3.63, *p* = 0.06	

**Table 3:** Estimated marginal means and 95% confidence interval (95% C.I.) of clinical outcomes for the two-study groups at the baseline and the two follow up visits.

Outcome	Baseline (T_0_)		Follow-Up (T_1_)		Time and Intervention Effects (T_1_ vs. T_0_)	Follow-Up (T_2_)		Time and Intervention Effects (T_2_ vs. T_0_)	Overall Effect of Intervention *p*-Value
	Control (n = 161)	Intervention (n = 142)	Control (n = 161)	Intervention (n = 142)		Control (n = 161)	Intervention (n = 142)		
**Plaques index (%)**	80.48 (76.04–85.17)	77.17 (71.99–82.73)	72.31 (67.93–76.98)	69.02 (64.18–74.22)	Time Z = 9.35, *p* = 0.002	79.06 (75.21–83.10)	72.95 (68.33–77.88)	Time Z = 0.31, *p* = 0.58	0.36
					Intervention× Time Z = 0.06, *p* = 0.93			Intervention × Time Z = 0.59, *p* = 0.44	
**Proportion of subjects with PPD ≥ 5 mm**	51.95 (44.07–59.73)	52.94 (44.55–61.17)	34.42 (27.34–42.25)	36.03 (28.41–44.42)	Time Z = 13.89, *p* < 0.001	43.51 (35.90–51.43)	37.50 (29.78–45.92)	Time Z = 2.99, *p* = 0.08	0.87
					Intervention × Time Z = 0.01, *p* = 0.92			Intervention × Time Z = 0.95, *p*-value = 0.33	
**Proportion of sites with BOP**	31.65 (27.24–36.78)	36.18 (31.31–41.81)	23.04 (20.05–26.48)	24.46 (21.37–27.99)	Time Z = 13.06, *p* < 0.001	26.53 (24.13-29.28)	26.74 (23.84–29.99)	Time Z = 4.36, *p* = 0.04	0.21
					Intervention × Time Z = 0.33, *p* = 0.56			Intervention × Time Z = 1.19, *p* = 0.28	

PPD; Periodontal Probing Depth and BOP; Bleeding on Probing.

## Data Availability

The data presented in this study are available on request from the corresponding author.
